# Facial Weakness Revealing Rare Familial Polyneuropathy

**DOI:** 10.1002/ana.78318

**Published:** 2026-07-30

**Authors:** Sanem Pinar Uysal, Ciersten A. Burks, Rex N. Smith, Paloma Gonzalez-Perez

**Affiliations:** 1 Department of Neurology, Division of Neuromuscular Medicine, Mass General Brigham, Harvard Medical School, Boston, MA, USA; 2 Department of Otolaryngology-Head and Neck Surgery, Division of Facial Plastic and Reconstructive Surgery, Mass Eye and Ear, Harvard Medical School, Boston, MA, USA; 3 Department of Pathology, Mass General Brigham, Harvard Medical School, Boston, MA, USA

A 56-year-old man developed progressive difficulties with smiling and raising eyebrows, an increasingly hooded appearance of his eyes and slurred speech around the age of 40 years. He was unable to retain solid food or liquids in his mouth but denied any difficulty with swallowing or chewing. He eventually also developed bilateral hand dexterity impairment and paresthesia in his fingertips. He denied diplopia or breathing difficulties. He also denied syncopal episodes, early satiety, impaired sweating, or temperature intolerance. He had no bowel or bladder symptoms. His medical history included myocardial infarction at the age of 41 years, corneal lattice dystrophy, hypertension, and hypercholesterolemia. He had Finnish ancestors and similar symptoms developed in prior generations on his paternal side.

His examination revealed pronounced sagging of skin (A) resulting in cutis laxa affecting the face (B) and arm (C), and facial weakness leading to inability to smile (D) or raise eyebrows (E). There was also severe bilateral facial weakness on eye and lip closure, and cheek puff. He had decreased hearing in the right ear to finger rubbing. Vibratory sensation was absent at the toes and ankles. Pinprick sensation revealed hyperalgesia in both feet and was decreased in the thumb, index, and middle fingers of both hands. He had normal deep tendon reflexes throughout, and gait and coordination were normal. Nerve conduction studies demonstrated mild bilateral carpal tunnel syndrome, left ulnar neuropathy at the elbow, and mild length-dependent axonal sensory polyneuropathy. Concentric needle electromyography of the right frontalis and right tongue muscles demonstrated normal insertional activity and reduced recruitment of long-duration and high-amplitude motor unit potentials. A pathogenic heterozygous variant (c.640G>A, p.Asp214Asn) within the gelsolin (*GSN*) gene was identified and a diagnosis of gelsolin amyloidosis (GA) was confirmed. This is the most common pathogenic variant in GA and has been associated with a gain-of-function mechanism that results in the aggregation and deposition of insoluble, polymerized gelsolin fragments in various organs.^1^

He underwent bilateral, minimally invasive brow lift and upper blepharoplasty to ameliorate the hooded appearance of his eyes (F). Histopathological analysis of the skin revealed abundant amyloid deposits on Congo red staining and apple-green birefringence under polarized light. Electron microscopy revealed ultrastructural amyloid fibrils measuring 9 nanometers (G–I). Mass spectrometry revealed moderately elevated gelsolin levels and keratin-type amyloid deposition. The healing process after the biopsy was uneventful and without complications (Figure).

Although phenotypic variability within and between families occurs, corneal lattice dystrophy within the third or fourth decade of life is often the first manifestation of GA.^2^ This condition should increase suspicion for GA before other disease manifestations, such as facial muscle weakness, cutis laxa, or potentially life-threatening cardiac arrhythmias, develop.^3^ Unlike other types of amyloidosis, autonomic involvement in GA is uncommon and milder when present.^4^

GA is a late-onset, non-length-dependent, familial polyneuropathy that, although rare, requires prompt recognition to avoid delays in diagnosis and unnecessary testing, establishing appropriate medical management such as cardiac surveillance and surgical management when indicated, and providing genetic counseling for patients and families.

## Figures and Tables

**FIGURE:**
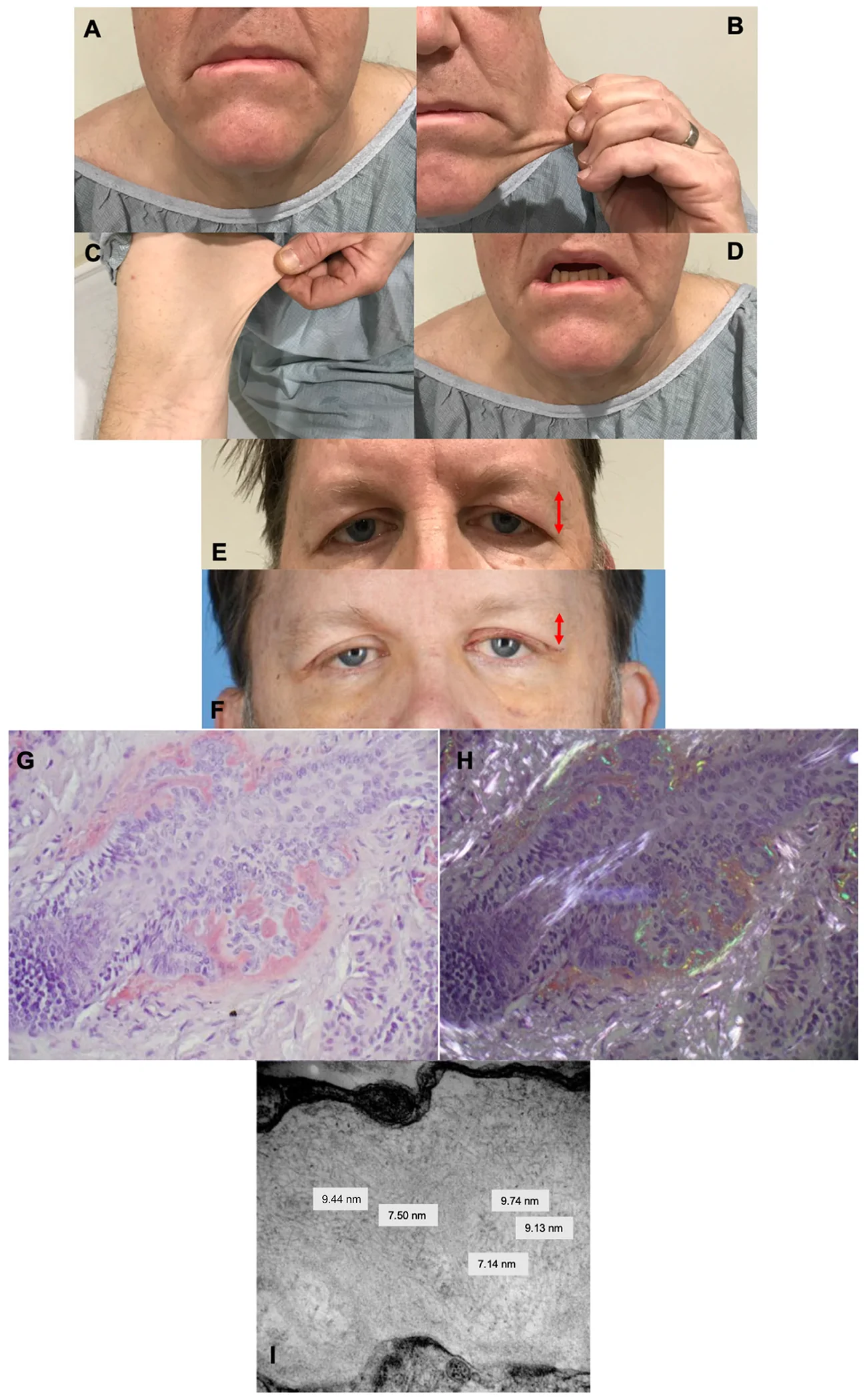
Phenotypic features and histopathological analyses of skin following blepharoplasty: Sagging of skin (A) resulting in cutis laxa affecting face (B) and arm (C). Facial weakness leading to inability to smile (D) or raise eyebrows (E). Improvement in appearance of hooded eyes following surgery (F). Congo red stain showed amyloid deposition circumferentially located around Meibomian glands and follicles (G). Same specimen demonstrated apple-green birefringence under polarized light confirming amyloid deposition (H). Electron microscopy revealed ultrastructural amyloid fibrils of 9 nanometers corresponding to gelsolin deposition (I).

## Data Availability

The data used during the current study is available from the corresponding author upon reasonable request.
